# In vivo 3D high resolution cardiac diffusion weighted MRI: a motion compensated diffusion-prepared balanced steady-state free precession approach

**DOI:** 10.1186/1532-429X-16-S1-O83

**Published:** 2014-01-16

**Authors:** Christopher T Nguyen, Zhaoyang Fan, Behzad Sharif, Yi He, Rohan Dharmakumar, Daniel S Berman, Debiao Li

**Affiliations:** 1 Biomedical Imaging Research Institute, Cedars-Sinai Medical Center, Los Angeles, California, USA; 2 Bioengineering, University of California Los Angeles, Los Angeles, California, USA; 3 Radiology, Anzhen, Beijing, China

## Background

Cardiac diffusion-weighted MRI has the potential to identify acute myocardial infarction, myocarditis, and myocardial fibrosis [1-3]. The aim of this study was to implement and optimize a novel application of diffusion-prepared bSSFP to perform in vivo cardiac diffusion-weighted MRI.

## Methods

Diffusion-prepared sequences have the flexibility to diffusion encode with a multi-shot image readout. The diffusion preparation was optimized to reduce sensitivity to cardiac bulk motion with second order motion compensation (M1M2). The image readout consists of a 3D centric phase encoded segmented bSSFP acquisition that incorporates a prospective navigator. Ten healthy subjects were scanned twice (once in the beginning and at the end) on a 1.5T system (Siemens Avanto) using the proposed technique (TR/TE = 3.4/1.3 ms, FOV = 256 × 256 mm^2^, α = 110°, 160 × 160 matrix, 10 mm slice thickness, 4 slices with 20% oversampling, 40 mm 3D slab, 5 linear ramp-up, b = 450 s/mm^2^, G_diff _= 40 mT/m). Diffusion preparation was applied in the diastolic phase with (TEprep = 115 ms) and without (TEprep = 45 ms) M1M2 using 3 orthogonal directions under varying off-resonance conditions. Trace apparent diffusion coefficient (trADC) maps and the left ventricular (LV) trADC were calculated. For each slice, the LV was segmented into six AHA segments. Statistical significance was tested using two tailed paired t-test for two mean comparisons and one-way ANOVA for multiple means comparisons.

## Results

M1M2 diffusion-prepared scans resulted in LV trADC values of 1.5 ± 0.4 × 10^-3 ^mm^2^/s that were reproducible yielding no statistical differences (p = 0.54). Regional differences between six AHA segments were not statistically significant across all subjects (p = 0.97). M1M2 diffusion-prepared images showed no ghosting artifacts and/or signal fallout. Under certain substantial off-resonance frequencies (e.g +200 Hz), the proposed method failed in yielding both T2prep and DW images when bSSFP-related banding formed. The non-motion compensated diffusion-prepared scans yielded LV trADC values of 6.6 ± 0.9 × 10^-3 ^mm^2^/s and diffusion-prepared images with severe bulk motion-induced artifacts.

## Conclusions

The LV trADC values derived with M1M2 motion compensation diffusion preparation were consistent with previously reported values ranging from 0.8 to 2.4 × 10^-3^mm^2^/s [4-6]. The uncompensated diffusion preparation measurements yielded LV trADC values that were much greater suggesting motion corruption. We developed a novel free-breathing bulk motion compensated diffusion-prepared 3D segmented bSSFP technique able to perform in-vivo cardiac diffusion-weighted MRI on a clinical MR scanner.

## Funding

NIH/NHLBI RO1 HL38698.

**Figure 1:**
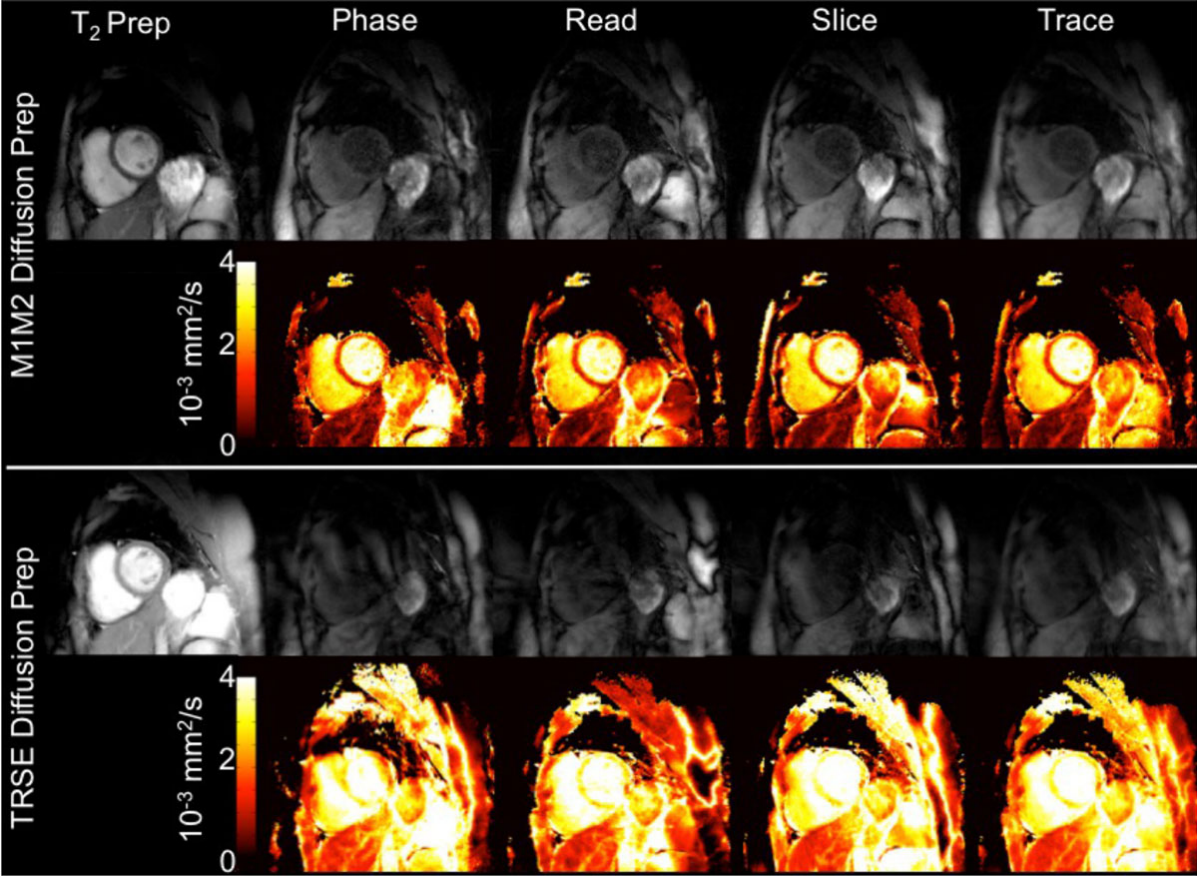
**Typical T2 prepared (b = 0 s/mm^2^) images, DWI (b = 450 s/mm^2^) in three orthogonal directions, and ADC maps for M1M2 (top) and TRSE (bottom) diffusion preparation**. M1M2 diffusion-prepared images clearly depict myocardium with minimal artifacts and yield reasonable myocardial trADC values. TRSE diffusion-prepared images present ghosting artifacts and signal drop out that are expected to arise in bulk motion corrupted diffusion-prepared sequences. LV trADC value derived from TRSE diffusion preparation are beyond diffusion of free water at 37°C (3.1 × 10^-3 ^mm^2^/s).

**Figure 2:**
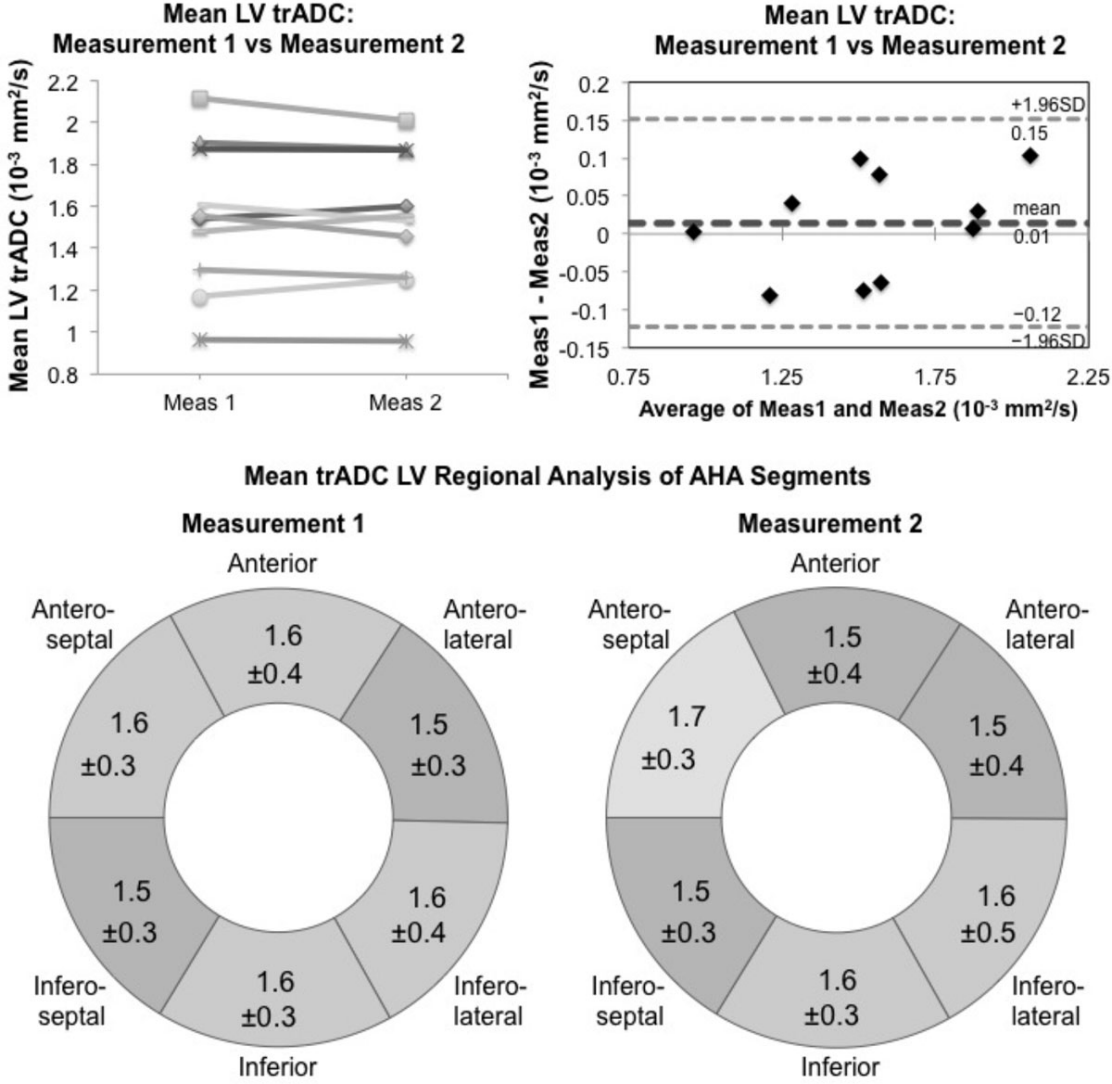
**(Top) Reproducibility of the proposed M1M2 diffusion-prepared bSSFP**. (Top Left), Line plot of the intrastudy reproducibility between measurement 1 and 2 of the LV trADC values displays consistency in measurements. (Top Right) Bland-Altman plot depicts no apparent bias and good agreement between the two measurements. No statistical differences were found between the two measurements. (Bottom) AHA wheel diagrams for LV regional analysis depict fairly homogenous mean trADC values (units: 10^-3 ^mm^2^/s) for both measurements 1 and 2. Additionally, the SD for each segment was calculated across all subjects. One-way ANOVA shows no statistically significant differences across all subjects for both measurements (p < 0.05 not satisfied).
